# Developmental Neurotoxicants in E-Waste: An Emerging Health Concern

**DOI:** 10.1289/ehp.1002452

**Published:** 2010-11-15

**Authors:** Aimin Chen, Kim N. Dietrich, Xia Huo, Shuk-mei Ho

**Affiliations:** 1 Department of Environmental Health, University of Cincinnati College of Medicine, Cincinnati, Ohio, USA; 2 Analytical Cytology Laboratory and Key Immunopathology Laboratory of Guangdong Province, Shantou University Medical College, Shantou, Guangdong, People’s Republic of China

**Keywords:** cadmium, chromium, developmental neurotoxicity, epigenetics, e-waste, lead, mercury, polybrominated diphenyl ethers, toxicologic mechanisms

## Abstract

**Objective:**

Electronic waste (e-waste) has been an emerging environmental health issue in both developed and developing countries, but its current management practice may result in unintended developmental neurotoxicity in vulnerable populations. To provide updated information about the scope of the issue, presence of known and suspected neurotoxicants, toxicologic mechanisms, and current data gaps, we conducted this literature review.

**Data sources:**

We reviewed original articles and review papers in PubMed and Web of Science regarding e-waste toxicants and their potential developmental neurotoxicity. We also searched published reports of intergovernmental and governmental agencies and nongovernmental organizations on e-waste production and management practice.

**Data extraction:**

We focused on the potential exposure to e-waste toxicants in vulnerable populations—that is, pregnant women and developing children—and neurodevelopmental outcomes. In addition, we summarize experimental evidence of developmental neurotoxicity and mechanisms.

**Data synthesis:**

In developing countries where most informal and primitive e-waste recycling occurs, environmental exposure to lead, cadmium, chromium, polybrominated diphenyl ethers, polychlorinated biphenyls, and polycyclic aromatic hydrocarbons is prevalent at high concentrations in pregnant women and young children. Developmental neurotoxicity is a serious concern in these regions, but human studies of adverse effects and potential mechanisms are scarce. The unprecedented mixture of exposure to heavy metals and persistent organic pollutants warrants further studies and necessitates effective pollution control measures.

**Conclusions:**

Pregnant women and young children living close to informal e-waste recycling sites are at risk of possible perturbations of fetus and child neurodevelopment.

Electronic waste (e-waste) has emerged as a critical global environmental health issue because of its massive production volume and insufficient management policy in many countries (Ogunseitan et al. 2009). E-waste includes waste cathode ray tube (CRT) televisions, desktops, laptops, CRT monitors, liquid crystal display (LCD) monitors, cell phones, keyboards, computer mice, printers, and copiers. E-waste contains metals and persistent organic pollutants (POPs); inappropriate recycling processes occur in several developing countries and result in the release of these toxicants into the environment (LaDou and Lovegrove 2008; Robinson 2009). Although serious health concerns arise from these primitive recycling activities, the research needs are largely unaddressed. The developing fetus and child are particularly vulnerable to several known and suspected developmental neurotoxicants in e-waste.

In this review, we survey the literature to provide updated information about major toxicants in e-waste, potential neurodevelopmental toxicity in children, and potential preventative measures to reduce exposure. Because the rate of e-waste accumulation is startling and the combinatorial effects of toxicants are complex, this review addresses an urgent need to evaluate potential adverse health effects of this unprecedented exposure scenario.

## Production and Management of E-Waste

**Figure 1:**
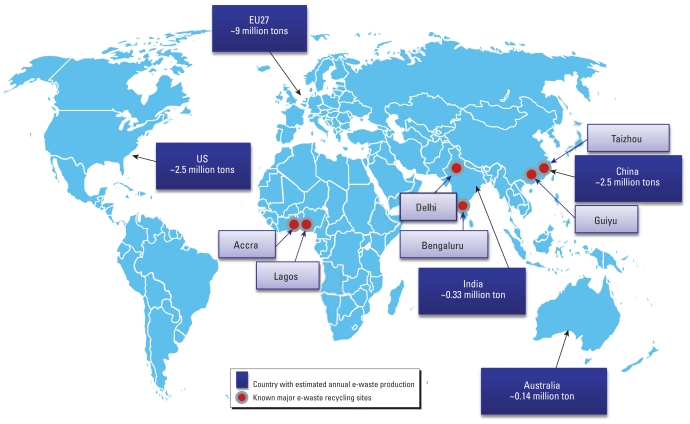
Estimated annual production of e-waste and major recycling sites. Estimates are from Robinson (2009), Davis and Herat (2010), and Cobbing (2008) and may not reflect current production. In addition, the estimates are not complete for many regions, for example, Japan, Russia, and Canada. The number of recycling sites is by no means complete but may represent major processing regions of e-waste.

E-waste is the fastest-growing stream of municipal solid waste, but its management is a significant environmental health concern. It is estimated that 20–50 million tons of e-waste are produced annually worldwide; the United States, Western Europe, China, Japan, and Australia are the major producers [Cobbing 2008; Davis and Herat 2010; Robinson 2009; United Nations Environment Programme (UNEP) 2005]. Figure 1 shows an incomplete list of e-waste volume and major informal recycling sites. According to a U.S. Environmental Protection Agency (EPA) estimate, the United States generated approximately 2.5 million tons of e-waste in 2007, which accounts for about 2% of municipal solid waste and has a projected annual increase of 3–5% (U.S. EPA 2008). In the United States, only about 18% of e-waste is collected for recycling, with the remaining 80% sent to landfill and 2% for incineration ([Bibr b107-ehp-119-431][Bibr b105-ehp-119-431]). Landfill can cause metal leaching from the e-waste (Dagan et al. 2007). Burning e-waste may produce extremely toxic dioxins and furans (Li et al. 2007). Environmentally friendly recycling has not been widely used, although it is a promising approach to tackle the e-waste problem (UNEP 2009). The European Union has enacted two directives to address the increasing concerns on e-waste: the Restriction on the Use of Hazardous Substances (RoHS) and the Waste Electrical and Electronic Equipment (WEEE) (European Union 1995, 1996; LaDou and Lovegrove 2008; Ogunseitan et al. 2009). The RoHS directive restricts the use of lead (Pb), cadmium (Cd), mercury (Hg), hexavalent chromium [Cr(VI)], polybrominated biphenyls, and polybrominated diphenyl ethers (PBDEs) in new electronic devices. The WEEE directive requires the manufacturers to take responsibility for collecting and recycling (“take-back”) of the e-waste. In contrast, the United States does not have legally enforceable federal policies to regulate e-waste despite a patchwork of legislation in about 25 states (e.g., mandating statewide e-waste recycling or banning landfill disposal of CRT monitors) [Electronics Take Back Coalition (ETBC) 2010; LaDou and Lovegrove 2008; Ogunseitan et al. 2009]. These include most coastal states, the Great Lakes states, Oklahoma, and Texas. Japan has an existing recycling system for limited home electrical appliances, but it does not cover all e-waste, and illegal dumping and transfer still occur (Aizawa et al. 2008; LaDou and Lovegrove 2008).

Although the Basel Convention regulates transboundary movement of hazardous waste, significant amounts of e-waste have been exported to developing countries and recycled in local towns and villages, using primitive technologies (LaDou and Lovegrove 2008). In the biomedical literature, primitive recycling of e-waste occurs in Guiyu, Taizhou, and Jinghai, China (Huo et al. 2007; Wong et al. 2007), Bengaluru and Dehli, India (Chatterjee 2008; Ha et al. 2009), Lagos, Nigeria (Osibanjo and Nnorom 2007; Schmidt 2006), and Trang Minh, Dong Mai, and Bui Dau, Vietnam (Tue et al. 2010) (see also Figure 1). A recent report listed a few other countries that may have small-scale informal e-waste recycling (Brazil, Colombia, Kenya, Mexico, Morocco, Peru, Senegal, South Africa, and Uganda) (UNEP 2009). Developing countries are generating more and more e-waste in their own territories and may also feed the recycling business (LaDou and Lovegrove 2008; Robinson 2009). The purpose of recycling activities in these developing countries is to recover gold, silver, copper, zinc, iron, tin, and other metals for profit (Huo et al. 2007; Wong et al. 2007). However, because of a lack of stringent environmental regulation and worker protection, toxicants in e-waste cause serious contaminations of local air, dust, soil, and water (Ogunseitan et al. 2009; Schmidt 2006; Wong et al. 2007). The environmental consequence is dire in these regions if the activities remain uncontrolled. Further, informal recycling processes (dismantling, cutting, heating, acid leaching, and burning) in small town and village workshops expose the workers and residents to dangerous mixtures of metals and other pollutants (LaDou and Lovegrove 2008).

## Developmental Neurotoxicants in E-Waste

Electronic devices consist of a large number of chemical elements and compounds. Even a cell phone can contain > 40 elements from the periodic table (UNEP 2009). The metals in e-waste include steel (iron), copper, aluminum, tin, Pb, nickel, silver, gold, arsenic, Cd, Cr, indium, Hg, ruthenium, selenium, vanadium, and zinc. The toxicity of these chemicals in e-waste remains to be determined. However, some chemicals are known or suspected to have developmental neurotoxicity. Neurodevelopmental deficits are a serious concern of exposure to e-waste toxicants, because children living in e-waste recycling communities may have been exposed to high-level toxicant mixtures throughout their lifetime. Infants and young children have relatively smaller body weight than adults, but their toxicant body load can be higher because they have relatively low body weight [American Academy of Pediatrics (AAP) 2003]. Developing fetuses and young children are at critical windows of neuronal growth, differentiation, migration, synaptogenesis, and myelination. Disruption of these fine-tuned processes in human neurodevelopment can have detrimental effects (Dietrich 2010). The commonly assessed neurodevelopmental end points include intelligence quotient (IQ), memory, language, gross and fine motor skills, attention, executive functions, and behavior. Obviously, a focus on developmental neurotoxicity in this review does not exclude the possibility of adverse effects on other organ systems, but many previous human studies of metal and POP exposure in community settings revealed deficits in neurological functions in children (Dietrich 2010; Wright and Baccarelli 2007). Table 1 summarizes the developmental neurotoxicity and exposure routes of common e-waste toxicants. In addition to exposure to e-waste, children are exposed to these toxicants from other existing sources (e.g., Pb and Hg from power plants and other industrial emissions as well as diet) (AAP 2003).

### Lead

Pb is arguably the most-studied developmental neurotoxicant and unfortunately is also one of the major toxicants in e-waste. An old CRT television contains about 1.5–2 kg Pb, and a CRT computer monitor contains about 0.5 kg Pb ([Bibr b104-ehp-119-431][Bibr b105-ehp-119-431]). Pb has also been used in solder in printed circuit boards and other components (Ramesh et al. 2007). In 1- to 6-year-old children living in a primitive e-waste recycling site, the mean blood Pb level approaches 15 μg/dL, which is 50% higher than the neighboring control site (~ 10 μg/dL) (Huo et al. 2007; Zheng et al. 2008). Blood Pb levels ≥ 10 μg/dL in early childhood are detrimental to neurodevelopment, and the recognized adverse effects include impaired cognitive function, behavioral disturbances, attention deficits, hyperactivity, and conduct problems (Bellinger 2004). Newly identified neuroanatomical changes in young adults who are exposed to Pb in childhood include reduced gray matter in the prefrontal region and diffusivity changes in white matter that indicate effects on myelination and axonal integrity (Brubaker et al. 2009; Cecil et al. 2008). Childhood Pb exposure, especially early-school-age blood Pb levels, strongly predict neurologic deficits in children and young adults (Hornung et al. 2009). There is a considerable amount of evidence showing that every 10-μg/dL increase of blood Pb concentration is associated with a deficit of 2–3 IQ points (Pocock et al. 1994). Current research suggests that a blood Pb concentration < 10 μg/dL is also harmful for cognitive function (Canfield et al. 2003; Lanphear et al. 2005). High Pb exposure in childhood has been associated with delinquent behaviors and criminal activities in adolescents and young adults (Needleman et al. 2002; Wright et al. 2008). In children, Pb exposure has also been associated with increased risk of attention deficit hyperactivity disorder (Braun et al. 2006). E-waste exposure represents a situation of continuous exposure, which raises concerns about neurodevelopmental deficits in young children and across the lifespan.

### Mercury

Because Hg is used in laptop monitors, cold cathode fluorescent lamps, cell phones, and printed circuit boards (e.g., switches, relays), improper recycling of e-waste may release Hg in its elemental vapor form into the environment (Ramesh et al. 2007). Each individual electronic device contains a very small amount of Hg (< 1–2 g) (U.S. EPA 2007), but intensive processing of millions of these devices could be highly problematic for the environment. In bodies of water, bacteria can transform inorganic Hg to organic form [i.e., methylmercury (MeHg)], and fish bioaccumulate MeHg. Eating MeHg-contaminated fish is the primary route of exposure in the general population, but people living in e-waste recycling sites may be exposed to both inorganic and organic Hg. So far, there is a lack of studies investigating Hg levels in children who lived in e-waste recycling sites. Currently, there is considerable debate about neurodevelopmental effects of moderate MeHg levels (maternal hair Hg 4–6 μg/g) or lower-level exposure because of conflicting results from the research in the Faroe Islands and Seychelles (Debes et al. 2006; Grandjean et al. 1997; Myers et al. 2009). The study in the Faroe Islands identified an association of prenatal MeHg exposure [geometric mean (GM) = 4 μg/g in maternal hair and 23 μg/L in cord blood] and deficits in motor function, attention, and verbal domains in children up to 14 years of age but did not find associations for postnatal exposure (GM = 3 μg/g in hair and 9 μg/L in blood at 7 years of age) (Debes et al. 2006). The research in Seychelles, however, did not find a consistent pattern of association between prenatal (mean = 7 μg/g in maternal hair) or postnatal MeHg (mean = 6 μg/g in hair at 9 years of age) and neurodevelopmental end points (Davidson et al. 2010; Myers et al. 2009). An integrative analysis of three cohorts (Faroe island, Seychelles, and another New Zealand study) found an overall child IQ change of −0.18 points [95% confidence interval (CI), −0.38 to −0.01] for each microgram per gram increase of maternal hair MeHg (Axelrad et al. 2007). A recent study of U.S. background level MeHg exposure (~ 0.5 μg/L in whole blood at 2 years of age) did not reveal significant associations with neurodevelopmental outcomes in children at ages 2, 5, and 7 years (Cao et al. 2010). For Hg vapor exposure from dental amalgam, two recent large clinical trials did not find adverse effects on cognitive function in children (Bellinger et al. 2006; DeRouen et al. 2006). In some Asian coastal regions, the GM hair Hg levels can reach 1–2 μg/g in women of reproductive age (Liu et al. 2008). The exposure to Hg from e-waste recycling needs to be characterized in pregnant women and young children from e-waste recycling sites, and if elevated, the neurodevelopmental effects should be examined.

### Cadmium

Cd is used in nickel–cadmium (Ni-Cd) batteries, surface mount devices chip resistors, infrared detectors, and semiconductor chips (Ramesh et al. 2007). Lithium-ion batteries have replaced Ni-Cd batteries in many electronic devices, but e-waste still contains old rechargeable batteries. Compared with Pb and Hg, the adverse neurodevelopmental effects of Cd are less well characterized in children. Cd levels in hair have been associated with deficits in cognition, learning, behavior, and neuromotor skills in children in earlier studies (Pihl and Parkes 1977; Thatcher et al. 1982), but inadequate control for Pb levels in the data analysis has been a concern. A recent study indicates that current background Cd exposure (~ 0.2 μg/L) in U.S. children may not cause significant cognitive and behavioral problems (Cao et al. 2009). In a Chinese birth cohort study, however, higher Cd exposure in cord blood (> 0.6 μg/L) was associated with a 4-point Full-Scale IQ deficit at preschool age after adjustment for cord blood Pb levels (Tian et al. 2009). The placenta limits the transfer of Cd from mother to fetus after the first trimester, but high cord blood Cd in infants may suggest high maternal exposure. These infants may also be postnatally exposed to Cd in the same maternal living environment (Osman et al. 2000). Because the half-life of Cd in kidneys and bones is estimated to be 10–30 years (Jarup and Akesson 2009), caution should be exercised to prevent Cd exposure in young children. The average blood Cd levels in children from an e-waste recycling site in China was 1.6 μg/L, significantly higher than the control site (1.0 μg/L) (Zheng et al. 2008). In Asian countries where rice consumption and environmental tobacco smoke are more common, children already get higher Cd exposure than those in Western countries (Jarup and Akesson 2009). Living in an e-waste recycling site substantially increases exposure of children to Cd, but the neurodevelopmental effects remain to be determined.

### Hexavalent chromium

Cr(VI) is used in metal coatings of some electronic devices for corrosion protection. It is a known human carcinogen after occupational inhalation exposure, but its toxicity in fetuses and children after environmental exposure is largely unknown (Pellerin and Booker 2000). Epidemiologic study of Cr exposure and child neurodevelopment is lacking. One animal study reported motor activity decrease in rats after chronic Cr exposure (az-Mayans et al. 1986). Oxidative stress in hypothalamus and anterior pituitary has been reported in Cr-exposed animals (Nudler et al. 2009). Increased urinary 8-hydroxy-2′-deoxyguanosine, a biomarker for oxidative DNA lesions, was reported in children with high urinary Cr (Wong et al. 2005). E-waste recycling can result in high Cr exposure in fetuses, with one report of mean cord blood Cr of 99 μg/L, significantly higher than the control-site mean of 32 μg/L (Li et al. 2008). The reported Cr levels were very high compared with findings from a large U.K. study (serum ~ 0.5 μg/L) and Italian Cr workers (whole blood ~ 6.9 μg/L) (Davies et al. 1997; Minoia and Cavalleri 1988).

### PBDEs

PBDEs—a group of brominated flame retardants—are used in electronic products to reduce flammability. Animal studies of PBDEs strongly suggest increased risk of thyroid hormone disruption [PBDEs and thyroxine (T_4_) are structurally similar], hyperactivity, cognitive deficits, and impaired memory (Costa and Giordano 2007). Susceptibility of children to PBDEs is a major concern, because children often have two to three times higher serum concentrations than their parents (Toms et al. 2009). A recent publication of prenatal exposures to PBDEs and Full-Scale IQ deficits [four points by interquartile range of BDE-47 (20 ng/g lipid)] in preschool children raised the concern of neurodevelopmental consequences (Herbstman et al. 2010). This association needs to be confirmed in other cohort studies. Median serum ∑BDEs (BDE-209 included) of up to 600 ng/g lipid were detected in recycling workers (Bi et al. 2007), but most studies found a mean or median of 100–400 ng/g lipid in the sera of local residents in the recycling sites (Qu et al. 2007; Yuan et al. 2008; Zhao et al. 2010). Breast milk from lactating mothers in the recycling sites also contains high PBDEs, with reported ∑BDEs of 84 ng/g (BDE-209 included) and 117 ng/g (BDE-209 not included) lipid in two different small studies in Vietnam and China, respectively (Leung et al. 2010; Tue et al. 2010). In contrast, the median serum ∑BDEs in the U.S. general population is ~ 40–60 ng/g lipid (BDE-209 not included), and that in Europe and Asia is usually < 10 ng/g lipid (Sjodin et al. 2008; Zhu et al. 2009). The e-waste recycling processes release significant amounts of BDE-209 that are not often detected in the U.S. population (Bi et al. 2007; Yuan et al. 2008). Although BDE-209 has a shorter half-life than less- brominated congeners in the environment, it may be degraded to the latter compounds and its toxicity remains to be determined (Birnbaum and Staskal 2004).

### Other toxicants: polychlorinated biphenyls, dioxins/furans, polycyclic aromatic hydrocarbons

Polychlorinated biphenyls (PCBs) were present in old transformers and capacitors before their ban in the 1970s, so e-waste recycling sites that deal with these devices may have high PCBs levels. Contemporary computers and cell phones do not contain PCBs. In Taizhou, China, where two sites are involved in e-waste recycling, Luqiao has higher serum ∑PCBs levels in adults (median = 118 ng/g lipid) than Wenling (median = 75 ng/g lipid), presumably because of a focus on PCB-containing devices and longer recycling history (Zhao et al. 2010). In another report, children in Luqiao have mean ∑PCBs of 222 ng/g lipid (boys) and 153 ng/g lipid (girls) (Ling et al. 2008). In contrast, adults in Guiyu, China, did not have higher ∑PCBs levels than a nonrecycling control site (median 52 vs. 63 ng/g lipid, respectively) (Bi et al. 2007), probably because Guiyu has been processing predominantly obsolete computers and cell phones.

Informal e-waste recycling also produces secondary emissions that are not chemicals in the e-waste but reaction products from incineration or smelting processes. Polychlorinated dibenzo-*p*-dioxins and dibenzofurans (PCDD/PCDFs) and polycyclic aromatic hydrocarbons (PAHs) can result from open burning of the e-waste (wires or plastics) to reduce volume or to recover metals. Even in primitive recycling sites, open burning of e-waste is usually prohibited. However, higher PCDD/PCDFs levels have been reported in breast milk, placenta, and hair samples from e-waste processing sites in Taizhou, China (Chan et al. 2007; Wen et al. 2008). The reported PCDD/PCDFs World Health Organization (WHO) toxicity equivalent (TEQ) level in breast milk was 21 pg/g lipid in Taizhou (Chan et al. 2007), twice as high as the levels in the United States and many European countries in a WHO-coordinated exposure study (van Leeuwen and Malisch 2002). No human studies of PAHs can be identified from e-waste recycling sites, but environmental samples (air, soil, and sediment) strongly suggest such contamination exists. The sum of 16 PAHs concentrations in PM_2.5_ (particulate matter with aerodynamic diameter ≤ 2.5 μm) air samples was 102 ng/m^3^ in a location close to an open burning site in Guiyu, China, much higher than average levels in Hong Kong (3–4 ng/m^3^) or Guangzhou (22–58 ng/m^3^) (Deng et al. 2006).

PCBs are known developmental neurotoxicants, and these compounds may affect a variety of neuropsychological functions in children, including general cognition, visual–spatial function, memory, attention, executive functions, and motor function (Boucher et al. 2009; Schantz et al. 2003). Most birth cohort studies of prenatal PCB exposure suggested a harmful role that was not accounted for by other environmental exposures, sociodemographic factors, child rearing, and parental IQ. The PCB levels in an e-waste recycling site (Taizhou) were in the low-to-moderate range of several international birth cohort studies of PCBs (using CB-153 as a criterion, median 30–450 ng/g lipid) (Longnecker et al. 2003). PCDD/PCDFs are often heat-degraded contaminants of PCBs; PCDFs in particular have been indicated to contribute to the two poisoning episodes in Japan and Taiwan (AAP 2003). The analytical testing of PCDD/PCDFs is more difficult, and thus epidemiologic studies are rare. However, in the Dutch PCB/dioxin study, lactational exposure to dioxin (median PCDD/Fs, WHO 1998 TEQ = 33 pg/g lipid in breast milk) was not associated with child cognitive abilities at 42 months of age (Patandin et al. 1999; Van den Berg et al. 2006). Similarly, a recent Duisburg birth cohort study in Germany (median PCDD/PCDFs) [WHO 2005 TEQ = 11 pg/g lipid in breast milk (Van den Berg et al. 2006)] did not find an inverse association with mental and psychomotor developmental indexes at 12 and 24 months of age (Van den Berg et al. 2006; Wilhelm et al. 2008). Additional studies are needed to investigate the neurodevelopmental effect of dioxins and furans at higher exposure levels. Recent studies have suggested that air pollutant PAHs may adversely affect child neurodevelopment and lead to IQ deficits (Edwards et al. 2010; Perera et al. 2009a; Tang et al. 2008). In the New York City and Polish studies, prenatal PAH exposure above the median (2.26 ng/m^3^ in New York City and 17.96 ng/m^3^ in Poland) was associated with an IQ deficit of about 3–5 points at 5 years of age (Edwards et al. 2010; Perera et al. 2009a).

## Unique Characteristics of E-Waste Toxicant Exposure

First, e-waste toxicants are released in uncontrolled recycling processes as a mixture. It is not uncommon that heavy metals and POPs coexist in the environment in recycling workshops and nearby neighborhoods. Second, the e-waste toxicant exposure is not homogeneous. The variability comes from several sources: the type of e-waste, length of recycling history, quantity of recycling, specialization in recycling processes, locations of workshops, parental involvement in recycling, and the daily activities of the child. Third, the exposure to e-waste toxicants lasts a long time. Many of the recycling sites have operated for more than a decade, and cumulative exposure in the local environment is typically high. Pregnant women who grew up in the recycling sites would have an even longer exposure history and higher body burden in physiologic deposits (e.g., bones and adipose tissues) than in women who moved in at the time of marriage. Transplacental and lactational exposure is expected for most metals and lipophilic organic pollutants in e-waste. Infants and children are exposed from contaminated indoor and outdoor air, dust, and soil. If the food and drinking water also come from contaminated community, the exposure will aggregate to a higher level.

## Potential Mechanisms of E-Waste Toxicants and Neurodevelopment

**Figure 2:**
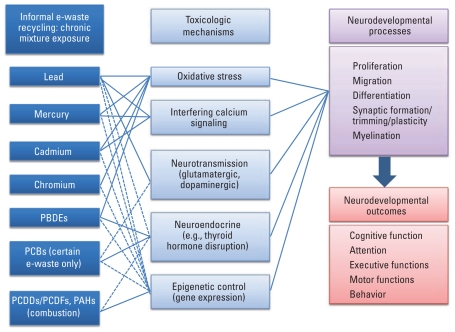
Potential developmental neurotoxicants in e-waste and their adverse effects on neurodevelopment in children. Solid lines represent more-studied links; dashed lines suggest possible links.

Toxicologic mechanisms of certain individual developmental neurotoxicants, especially Pb, have been investigated extensively, but data are insufficient to address exposure mixtures such as those in e-waste. Apparently several toxicologic mechanisms may be involved in this mixture of known and suspected neurotoxicants, but more research is needed to investigate the combinations of different metals and POPs (Figure 2). These toxicologic mechanisms are very complicated and may overlap, and other mechanisms related to molecular biology and signal transduction may be involved as well.

### Oxidative stress

Heavy metals can induce oxidative stress by increasing the production of reactive oxygen species (ROS) and depletion of antioxidant reserves (Wright and Baccarelli 2007). Neurons have limited capacity to detoxify ROS and are particularly vulnerable to oxidative stress. Pb exposure increases the formation of superoxide anion (^•^O_2_−) and hydrogen peroxide (H_2_O_2_) in the central nervous system (CNS), which may interact with proteins, lipids, and DNA to induce apoptosis (Sanders et al. 2009). MeHg affects the mitochondria electron transport system and causes overproduction of ROS (Johansson et al. 2007). Cd induces oxidative stress in cultured cells and animals and reduces antioxidant levels in humans (Joseph 2009; Lee et al. 2006). Exposure to PBDEs increase the generation of ROS, and different PBDEs congeners (e.g., BDE-47 and BDE-99) may have synergistic interactions in certain concentrations (Tagliaferri et al. 2010).

### Neurotransmission and calcium homeostasis

Many heavy metals can affect neurotransmission and disrupt the calcium-signaling pathway and thus interfere with synaptic functions. Pb ions (Pb2^+^) selectively bind *N*-methyl-d-aspartate (NMDA) receptor, one subtype of glutamatergic receptors (Toscano and Guilarte 2005). Glutamate is the major excitatory neurotransmitter in the brain tissues and is associated with learning and memory by the establishment of long-term potentiation (LTP). Interaction of Pb with the NMDA receptor increases Ca2^+^ influx, initiating cellular processes that lead to cell dysfunction (Sanders et al. 2009). MeHg exposure can increase Ca2^+^ levels in different cell types, and it may cause disruptions in cell cycles and migration (Johansson et al. 2007). Cd exposure may modify calcium channels and decrease the release of neurotransmitters glutamate and aspartate into the synaptic clefts (Minami et al. 2001). A recent animal study suggests that BDE-209 exposure reduces LTP and affects synaptic plasticity (Xing et al. 2009). The dopaminergic system is another critical CNS neurotransmission pathway that affects cognition, motivation and reward, attention, and learning. Extensive evidence on the role of environmental toxicants, such as Pb, on synaptic dopamine release, its receptors and transporters, and metabolism has emerged (Jones and Miller 2008).

### Neuroendocrine disruption

Previous research on heavy metals and neurotoxicity has suggested a similarity to the effects of subtle hypothyroidism, but the evidence is limited (Soldin et al. 2008; Wong et al. 1991). Thyroid-stimulating hormone (TSH), T_4_, and triiodothyronine (T_3_) could have unique effects on the initiation and modulation of gene expressions for brain development (Porterfield 2000). Animal studies have indicated potential disruption of transthyretin levels in the cerebrospinal fluid and brain deiodinase by Pb, Hg, or Cd (Mori et al. 2006; Soldin and Aschner 2007; Zheng et al. 2001). Pb exposure in occupational workers reduced total T_4_ (TT_4_), free T_4_ (FT_4_), or total T_3_ (TT_3_) (Lopez et al. 2000). Recent studies suggest a reduced level of FT_4_ associated with Pb exposure in pregnant women (Lamb et al. 2008) and in adolescents (Dundar et al. 2006), but the results are inconsistent (Maervoet et al. 2007; Schell et al. 2008). In one Canadian study, inorganic Hg was associated with a reduction of FT_4_ (Takser et al. 2005), but two other studies found nonsignificant associations in pregnant women and children (Osius et al. 1999; Schell et al. 2008). Cd exposure was found to affect TSH and FT_4_ levels in two recent studies (Iijima et al. 2007; Osius et al. 1999), but not in another (Maervoet et al. 2007). The animal studies of PCBs or PBDEs and thyroid hormone disruption have shown strong correlations, mostly reducing circulating T_4_ or T_3_ levels, but human studies are still needed to confirm the effects (Herbstman et al. 2008). A recent epidemiologic study of PBDEs suggests a slight decrease of TSH in exposed pregnant women (Chevrier et al. 2010). One study in e-waste recycling workers revealed higher TSH levels than in controls, and the role of PBDEs are suspected because of their structural similarity to T_4_ (Yuan et al. 2008). A recent larger study in e-waste recycling workers, however, found lower TSH levels than in controls (Wang et al. 2010). Another study suggested a lower TT_4_ level in maternal serum in relation to exposure to PCDD/PCDFs and PCBs (Zhang et al. 2010). Thyroid hormone alteration warrants further study in the exposure to e-waste toxicants.

### Epigenetic modifications

Epigenetic modifications are mitotically heritable changes of gene function in the absence of alterations in nucleotide sequence. These epigenetic changes include DNA methylation, mostly in the 5′-cytosine in the CpG dinucleotides of the gene promoter region, histone modifications, and microRNAs that affect posttranscriptional regulation (Baccarelli and Bollati 2009). Because nucleotide sequence is generally static in somatic cells and epigenetic markers are modifiable during the life course, the investigation of epigenetic changes induced by environmental toxicants has received increased attention (Baccarelli and Bollati 2009). Although examinations of these mechanisms in human neurodevelopmental studies are rare, several *in vitro*, *in vivo*, and human studies suggest possible perturbations in DNA methylation and histone modifications by Pb, Hg, Cd, Cr, PAHs, and PBDEs (Benbrahim-Tallaa et al. 2007; Chen et al. 2010; Jiang et al. 2008; Kondo et al. 2006; Onishchenko et al. 2008; Perera et al. 2009b; Pilsner et al. 2009, 2010; Sun et al. 2009; Takiguchi et al. 2003) (summarized in Table 2). Epigenetic changes may affect gene expression in specific tissues (e.g., brain regions) and subsequently modify neurodevelopment in a critical window of development, but the role of neurotoxicants needs to be determined.

## Data Gaps in E-Waste Toxicants and Developmental Neurotoxicity

### Lack of comprehensive exposure assessment

Comprehensive exposure assessment is urgently needed to characterize the profiles of chemicals and their concentrations, especially in countries where informal e-waste recycling exists on a large scale but exposure assessment is scarce, for example, in India (Ha et al. 2009). Exposure assessment should include both environmental and biological sampling in the recycling sites and control sites to determine the extent of exposure. Exposure of children needs to be examined from *in utero* to adolescence—in pregnant women (blood, urine, hair), neonates (meconium, cord blood, breast milk), and children (blood, urine, hair). Toxicant profiles including metals and POPs should be determined in the same study subjects to reflect a real-world exposure scenario of e-waste recycling.

### Lack of evaluation of adverse developmental effects

The demonstration of adverse health effects has historically preceded effective pollution control measures. Even though the precautionary principle has gained increasing attention in developed countries, in developing countries observed detrimental health effects are more likely to be a turning point in public opinion and policy making. Research is clearly needed to investigate the health effects of e-waste toxicants resulting from informal recycling activities. These health effects may include fetal development (birth weight, birth length, head circumference, gestational age, thyroid function) and child growth and neurodevelopment (cognition, memory, learning, motor function, executive functions, behavior).

### Lack of toxicologic mechanistic research

E-waste toxicant mixtures have not been examined for their potential mechanisms of human developmental toxicity. Even in *in vitro* or *in vivo* studies, investigation of mixture toxicity is rare. In the case of primitive e-waste recycling, there is a need—and indeed a unique opportunity—to integrate human exposure assessment, adverse health effects, and toxicologic mechanisms, because such exposure is unprecedented and complex. Mechanistic research that involves new advancements in genomics, epigenetics, and proteomics may provide novel understanding of these known and suspected neurotoxicants. Potential effect modification and synergistic interactions of these toxicants can also be determined in this complex exposure. Mechanistic research can also elucidate the pathway from exposure to internal dose and to biological markers of early adverse effects. Biomarkers including biochemical and epigenetic changes can be reliably assessed in various biospecimens such as blood, urine, buccal swab, and saliva. Integrating mechanistic research into human studies will supplement the findings of animal studies with direct evidence of modifiable molecular changes in exposed populations.

### Lack of investigation of preventive measures

Although informal e-waste recycling has occurred in developing countries for more than a decade, and high toxicant exposures in vulnerable population have been reported, few attempts have been made to intervene and reduce exposures in the local communities. Research can further determine the major contributing factors to high toxicant exposures that can be prevented or mitigated. Such factors could include locations of recycling workshops relative to residential communities, using houses as recycling workshops or storage, specific recycling processes and technical procedures, lack of personal and environmental protection during recycling, lack of additional protection for pregnant women and young children, and nutritional and behavioral factors (iron and zinc deficiency, insufficient vitamin intake, environmental tobacco smoke). These risk factors can be reduced at either a personal or community level and could reduce the exposures and adverse health effects even if the recycling activities do not cease immediately.

## Perspectives

### Investigations

E-waste is an emerging issue in environmental health, and its potential significance is now being recognized by both scientists and policy makers. However, serious data gaps exist in the quantification of exposures and health effects. In communities where informal recycling occurs, biomonitoring of exposures, especially in vulnerable pregnant women and young children, provide critical information for epidemiologic investigations, environmental policy making, and informed plans for intervention. Studies that use sensitive neurodevelopmental end points are particularly important in this complex exposure. Other potential toxicities in humans—for example, cancer, respiratory diseases, reproductive functions, and renal effects—should also be examined.

### Prevention

A systematic approach guided by exposure assessment and health effect research is needed to prevent toxicant exposures in e-waste. Engineers, environmental scientists, and other professionals can participate in the research to minimize exposure to these toxicants. Restricting the use of toxic chemicals in manufacturing of electronic devices will surely be the upstream of prevention efforts. Appropriate recycling technologies should be the mainstay of e-waste recycling practices. Informal and primitive recycling practices need to be significantly reduced or eliminated. Exposure of children to excessive e-waste toxicants should be minimized at both household and community levels.

### Environmental health policies

Effective environmental regulations in e-waste management are needed to prevent excessive exposure to toxicants. Both developed and developing countries share joint responsibility in regulating electronic device manufacturing and e-waste transboundary movement. In countries where primitive recycling processes exist, human health, especially the health of children, needs to drive the regulation and management of recycling activities.

## Figures and Tables

**Table 1:** Characteristics of known and suspected neurotoxicants in e-waste and from its informal recycling processes.

Toxicant	Potentially affected neuropsychological functions in children	Transplacental exposure	Lactational exposure	Exposure route of childhood
Pb	Cognition (verbal and performance), fine and gross motor skills, memory, attention, executive function, hyperactivity, academic achievement, delinquent behavior	Yes	Yes	Air, dust, water, soil, leaded paint, leaded gasoline (if not banned)
Hg	Cognition, language, motor function, attention	Yes	Yes	Air, seafood, Hg vapor
Cd	Cognition	Limited	Yes	Air, dust, rice, vegetables, environmental tobacco smoke
Cr	Motor function (animal study only)	Yes	Yes	Air, dust, water
PBDEs	Cognition	Yes	Yes	Air, dust, food
PCBs	Cognition, visual–spatial function, memory, attention, impulse control, executive function, motor, behavior	Yes	Yes	Air, dust, seafood
PCDD/PCDFs	Cognition	Yes	Yes	Air, dust, soil, food
PAHs	Cognition	Yes	Yes	Air, dust, soil, food

**Table 2:** Potential epigenetic modifications by environmental toxicants in e-waste.

Toxicant	Reference	Species/tissue/cell	Epigenetic effects
Pb	Pilsner et al. 2009	Human cord blood leukocytes	Maternal exposure associated with global hypomethylation
Hg	Pilsner et al. 2010	Polar bear brain	Brain Hg associated with brain genomic DNA hypomethylation
	Onishchenko et al. 2008	Mice hippocampus	Hypermethylation in brain-derived neurotropic factor gene
Cd	Takiguchi et al. 2003	Rat liver cells	Initial DNA hypomethylation, subsequent DNA hypermethylation after prolonged exposure
	Benbrahim-Tallaa et al. 2007	Cd-transformed prostate epithelial cells	Genomic hypermethylation, hypermethylation in *RASSF1A* and *p16* genes
	Jiang et al. 2008	Human embryo lung fibroblast cells	DNA hypermethylation
Cr	Kondo et al. 2006	Human lung cancer	Hypermethylation in *p16* gene
	Sun et al. 2009	Human lung A549 cells	Increased global histone H3 lysine 9 (H3K9) and H3K4 di- and trimethylation, decreased H3K27 trimethylation and histone H3 arginine 2 (H3R2) dimethylation
PAHs	Perera et al. 2009b	Human cord blood leukocytes	Hypermethylation of *ACLS3* gene promoter region
PBDEs	Chen et al. 2010	Neonatal rat hippocampal neurons	Global DNA hypomethylation
